# Accurate surface ultraviolet radiation forecasting for clinical applications with deep neural network

**DOI:** 10.1038/s41598-021-84396-2

**Published:** 2021-03-03

**Authors:** R. Raksasat, P. Sri-iesaranusorn, J. Pemcharoen, P. Laiwarin, S. Buntoung, S. Janjai, E. Boontaveeyuwat, P. Asawanonda, S. Sriswasdi, E. Chuangsuwanich

**Affiliations:** 1 Department of Computer Engineering, Faculty of Engineering, Chulalongkorn University, Bangkok, Thailand; 2 Computational Molecular Biology Group, Faculty of Medicine, Chulalongkorn University, Bangkok, Thailand; 3 Division of Information Science, Nara Institute of Science and Technology, Nara, Japan; 4 Photodermatology Unit, Division of Dermatology, Department of Medicine, King Chulalongkorn Memorial Hospital and Faculty of Medicine, Chulalongkorn University, Bangkok, Thailand; 5 Department of Physics, Faculty of Science, Silpakorn University, Nakhon Pathom, Thailand; 6 Research Affairs, Faculty of Medicine, Chulalongkorn University, Bangkok, Thailand

**Keywords:** Skin diseases, Computer science, Atmospheric science

## Abstract

Exposure to appropriate doses of UV radiation provides enormously health and medical treatment benefits including psoriasis. Typical hospital-based phototherapy cabinets contain a bunch of artificial lamps, either broad-band (main emission spectrum 280–360 nm, maximum 320 nm), or narrow-band UV B irradiation (main emission spectrum 310–315 nm, maximum 311 nm). For patients who cannot access phototherapy centers, sunbathing, or heliotherapy, can be a safe and effective treatment alternative. However, as sunlight contains the full range of UV radiation (290–400 nm), careful sunbathing supervised by photodermatologist based on accurate UV radiation forecast is vital to minimize potential adverse effects. Here, using 10-year UV radiation data collected at Nakhon Pathom, Thailand, we developed a deep learning model for UV radiation prediction which achieves around 10% error for 24-h forecast and 13–16% error for 7-day up to 4-week forecast. Our approach can be extended to UV data from different geographical regions as well as various biological action spectra. This will become one of the key tools for developing national heliotherapy protocol in Thailand. Our model has been made available at https://github.com/cmb-chula/SurfUVNet.

## Introduction

Phototherapy using artificial light sources is one of the standard treatments for various skin conditions^1–3^. With established national guidelines and standard dosimetry protocols, hospital-based phototherapy provides safe and effective treatment for a wide variety of patients. However, many skin patients in Thailand still lack access to hospital-based phototherapy due to the limited number of phototherapy centers as well as shortage of qualified phototherapy practitioners across the country.

Heliotherapy, or phototherapy using natural sunlight, has been reported effective for treating diverse health issues^4–6^ and skin conditions^7–9^ since 1890s. Several clinical studies have also shown success outcomes of supervised heliotherapy in mostly European countries, including the Canary Islands, Spain, Helsinki, Finland and Davos, Switzerland^10–15^. Despite clear benefits of heliotherapy, a key issue that limits its effectiveness is the substantial variation in surface UV radiation throughout the year and time of day. Therefore, accurate estimates of UV radiation, in conjunction with treatment action spectrum and dosimetry, are essential for developing a safe and effective heliotherapy protocol in long-term use for a particular geographical region^16–18^. To date, a few studies have explored the prospect of quantitative heliotherapy planning based on UV radiation forecast^17,19^.

Prediction of surface UV radiation can roughly be categorized into three groups: modeling based on the physics of UV radiation, a hybrid between the physics and empirical techniques, and deep learning. Modeling approaches based on the physics of UV radiation calculate the amount of solar UV radiation that arrives at a certain location on Earth at a certain time mainly based on the Earth–Sun distance and the thickness of the Earth’s ozone layer^19–21^. This is also known as the clear-sky UV radiation. Then, to obtain the amount of radiation on the Earth’s surface, the clear-sky estimates are multiplied by factors such as Cloud Modification Factor^19,22^ to account for reflection and scattering of UV in the atmosphere. Hybrid approaches rely on Physics knowledge to define UV-related factors, such as total ozone column, zenith angle, and weather conditions, but incorporate numerical simulations and regressions to estimate the contribution of these factors to the amount of surface UV radiation in a data-driven manner^22–25^. In contrast, deep learning approaches attempt to predict surface UV radiation data directly from past observations with little to no constraint on how UV-related factors interact^26–29^. Although deep learning is effective for forecasting time series^30^ because of its ability to learn complex non-linear relationship between the input and output data, it requires a large amount of data to train, lacks interpretability, and does not perform well on new datasets with different distributions. It is expected that deep learning model for UV forecasting needs to be retrained for each geographical region. Recent works in the energy domain have successfully utilized recurrent neural network (RNN)^31^ architectures, such as Long Short-Term Memory (LSTM)^32^ and Gated Recurrent Unit (GRU)^33^, to predict solar photovoltaic power production^26,27,34–36^.

Study of surface UV radiation in Thailand^37,38^ showed that this region has sufficient UV radiation year-round, indicating that heliotherapy is a promising treatment alternative for skin patients in the country. In this work, we developed a deep learning model for surface UV radiation forecasting based on the encoder–decoder architecture^39^ and 10-year surface UV radiation data collected at Nakhon Pathom, Thailand (13.82° N, 100.04° E) from 2009 to 2019. The model requires only past UV radiation data as input and is able to predict antipsoriatic effective irradiance^17^ at 10-min intervals from 8 AM to 4 PM with about 10% error for 24-h forecast and 13–16% error for 7-day up to 4-week forecast. As the model’s performances are well within the acceptable error range of 10–25% indicated in broadband UVB phototherapy guidelines worldwide^1,40^, our work serves as a key step toward the establishment of the national heliotherapy protocol in Thailand.

## Methods

### Surface UV and weather data acquisition

**Figure 1:**
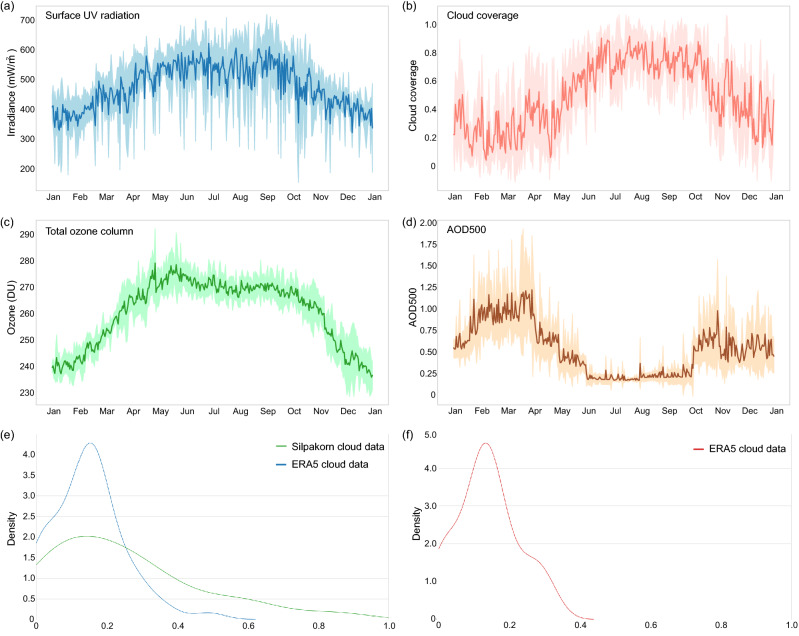
Characteristics of UV and weather conditions at Nakhon Pathom, Thailand. Daily maximums are shown for UV irradiance, total ozone column, and AOD500. Daily averages are shown for cloud coverage. Dark lines indicate the average across 2009–2017. Shaded areas indicate the ± 1 standard deviation range. (**a**) Annual surface UV irradiance. (**b**) Annual cloud coverage. (**c**) Annual total ozone column. (**d**) Annual AOD500. (**e**) The distribution of cloud coverage in the validation set (UV data from year 2018). Both Silpakorn University’s observations and ERA5 data were shown. (**f**) The distribution of cloud coverage in test set (UV data from year 2019). Information from Silpakorn University is unavailable.

Surface UV radiation, total ozone column, cloud coverage, and aerosol optical depth at 500 nm (AOD500), were collected at the Faculty of Science, Silpakorn University, Nakhon Pathom, Thailand (13.82° N, 100.04° E) from January 2009 to May 2019. UV intensity was measured every 10 min from 5AM to 7PM at 1-nm wavelength interval from 280 to 400 nm in mW/m^2^ unit using a DMc150 double monochromator (Bentham Instruments, Berkshire, UK). AOD500 and cloud coverage data were collected from 6 AM to 6 PM from January 2011 to December 2018. Hourly AOD500 data were measured by a ground based CE318 sunphotometer (Cimel Electronique, Paris, France) and calibrated by the Aerosol Robotic Network (NASA, Washington, DC, USA). Cloud coverage data were estimated on a 0–10 scale from recorded images of the sky every hour through a PSV-100 skyview instrument (Prede Company, Tokyo, Japan). Total ozone column data were measured daily in Dobson unit (DU) via an OMI/Aura satellite (NASA, Washington, DC, USA) from January 2011 to December 2019. The distributions of UV radiation, cloud, ozone and AOD500 in Nakhon Pathom throughout the year are shown in Fig. 1a–d, respectively.

Hourly downward surface UV radiation in J/m^2^, total ozone column in kg/m^2^, and mid cloud coverage were also downloaded from ERA5^41^ for London, England (51.5° N, 0° E) and Tokyo, Japan (35.75° N, 139.75° E) from 5AM to 7PM from January 2011 to December 2019. It should be noted that ERA5 datasets were generated from a combination of actual observation (every 3-h) and computational reanalysis. ERA5 downward UV radiation data cover the 200–440 nm wavelength range.

### Data cleaning and preprocessing

Surface UV radiation exhibits an annual seasonal pattern. We used this pattern as a justification for using UV data of the same dates from adjacent years to impute each missing data point. This is crucial because missing UV data often arise from sensor malfunction which typically spans multiple days. Also, because the artificial neural network model cannot handle missing values, imputation increases the number of data points that can be used to train and test the model. Specifically, we impute each missing data point with the average UV radiation from adjacent 10-min time steps, the same time steps from adjacent days, and the same dates from adjacent years. The ranges of adjacent time steps, days, and years that were used for imputation are 2, 5, and 2, respectively. Imputed data were visually inspected to ensure that the overall UV intensity follows the expected bell-shape pattern with a peak at around noon. In Thailand, this bell-shape pattern is often observed from October to January where there are few rainy and cloudy days. The Nakhon Pathom UV data from 2014 were excluded from further considerations as there is a technical problem with the instrument.

Nakhon Pathom UV data were split into a training set (2009–2017), for optimizing the parameters of artificial neural network models, a validation set (2018), for determining when to stop the optimization process, and a test set (2019), for evaluating the performance of the final models. We found that using the whole training set, i.e., using UV data from all dates and times, to train the models yielded the best performance. For the validation and test sets, we further exclude data from days with anomalous UV intensity profiles to prevent them from influencing the evaluation of the models. Specifically, we removed data from days whose UV profiles are highly skewed (absolute skewness greater than 0.3), disproportional (ratio between maximal and minimal irradiances greater than 15), or out of expected range (maximal irradiance above 400 or below 150 mW/m^2^). The distributions of cloud coverage in the validation and test datasets are shown in Fig. 1e and f, respectively. Finally, the antipsoriatic irradiance at each time point was calculated from 280 to 400 nm UV data based on published psoriasis clearance action spectrum formula^17,42,43^.

For evaluating the impact of incorporating ozone and AOD500 information as input into SurfUVNet, because these data were available only up to 2018, we re-split the dataset by setting data from 2009 to 2016 as the training set, data from 2017 as the validation set, and data from 2018 as the test set. The same quality filter for excluding data from days with poor UV profiles defined above was also applied to these validation and test sets. SurfUVNet model variants with and without ozone and AOD500 as input were then trained and evaluated together on this data split.

### SurfUVNet model architecture

**Figure 2:**
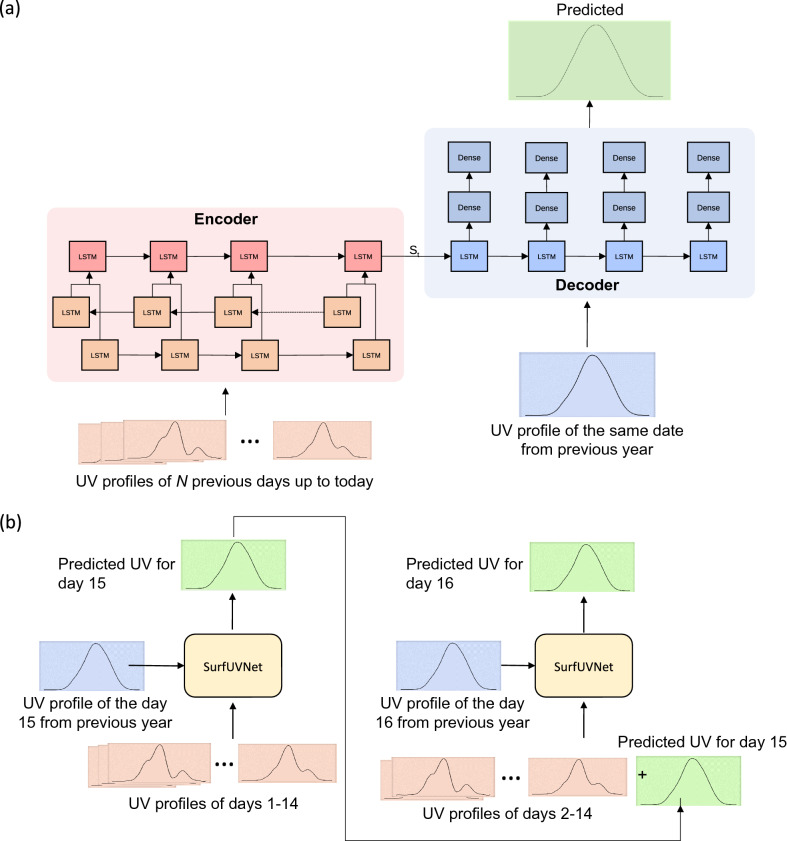
Schematic of SurfUVNet. (**a**) The underlying encoder–decoder neural network architecture showing the flow of data from the encoder to the decoder via the central connection denoted by S_t_. LSTM and Dense indicates the Long Short-Term Memory and fully connected neural network layers, respectively. UV data from days prior to the forecast date are fed into the encoder part while UV data from the same date of previous year are fed into the decoder part. The model forecasts next-day UV radiation at 10-min resolution. (**b**) The auto-recursive mode for long-term UV forecasting. To forecast UV radiation for the next *N* days, SurfUVNet first forecast next-day’s UV radiation profile and then uses the prediction as input to forecast UV radiation profile for the day after. This process is repeated until the forecasts for the next *N* days are generated.

Encoder–decoder-based model is a kind of deep learning model that have been successfully applied to various applications such as image captioning^44^ and machine translation^39^. In the context of UV forecasting, an encoder–decoder model can be used to translate a sequence of past observed UV radiations into a sequence of future UV radiations. The model consists of two parts: encoder and decoder as shown in Fig. 2a. Both parts consist of multilayered LSTMs. As the names implied, the LSTMs in the encoder are used for encoding information from the input sequence while the LSTMs in the decoder decoded that information to generate the output sequence.

As the input to our model, for the main implementation which relies only on UV data, we use a sequence of 10-min interval antipsoriatic data from the previous days, denoted as $\left[ {A_{1} , A_{2} , \ldots , A_{t} } \right]$ and a sequence of antipsoriatic data from the previous year, denoted as $\left[ {B_{1} ,B_{2} , \ldots ,B_{t} } \right]$. For the model variant which also accepts AOD500 and ozone, the inputs $A_{i}$’s and $B_{i}$’s will include these data of the same time-of-day from previous days and previous year as well. To handle differences in data resolution for various features (10-min for UV irradiance, hourly for AOD500, and daily for ozone), the values of features with lower resolutions were duplicated to match the highest resolution.

Since the antipsoriatic values are seasonal in nature, we also include day-of-year information as the input by encoding the day-of-year on a circular index defined as:

1 $$Circular\;Index\;Date = \left[ {sin\, 2\pi \left( { \frac{day }{{265}}} \right),\;cos\, 2\pi \left( { \frac{day }{{365}}} \right)} \right]$$

The circular date feature helps the model to learn the seasonal pattern. The model predicts future antipsoriatic values, $\left[ {\widetilde{y}_{1} , \widetilde{ y}_{2} , \ldots , \widetilde{ y}_{t} } \right]$.

Next, we provide detailed information of our model.

### Encoder

The decoder takes the previous day sequence $\left[ {A_{1} ,A_{2} , \ldots ,A_{t} } \right]$ and the circular date feature as input. We use a bi-directional^45^ LSTM as the first layer to help the model learns the temporal effect in both directions. The latter layers are uni-directional LSTM that will capture the information and pass the information to the decoder via the final cell state, $S_{t}$.

### Decoder

The decoder takes $\left[ {B_{1} ,B_{2} , \ldots ,B_{t} } \right]$ as input and uses it to future predict antipsoriatic values. The first layer of the decoder is a LSTM layer which uses $S_{t}$ from the decoder as the initial value of the cell state. We also add two fully connected layers with sigmoid activation function with the dropout^46^ rate of 0.2 after the LSTM layer for the final output.

Before feeding the input data to the model, we denoised the input antipsoriatic data with the Savitzky–Golay filter^47^. Applying the filter, smooth out the input data, removing any possible noise spikes in the data. However, we do not apply this processing to the target output data. If we train the model to predict the denoised data, the model is learning to predict the unrealistic data and will not be able to handle noises in the UV intensities. Then, we normalized the antipsoriatic data into a range of [0, 1].

We trained the model using quantile loss^48^ defined as:

2 $$L_{QUANTILE} = \frac{1}{N} \mathop \sum \limits_{i = 1}^{N} \max \left( {q\left( {\widetilde{y}_{i} - y_{i} } \right),\left( {q - 1} \right)\left( {\widetilde{y}_{i} - y_{i} } \right)} \right)$$

where $y_{i}$ is the actual value and $\widetilde{y}_{i}$ is the predicted value, *q* is a quantile value which balances the penalties of overestimates and underestimates. If *q* is more than 0.5, the quantile loss gives more penalty to overestimated predictions and vice versa. In our work, we set *q* to 0.33 to favor overestimation rather than underestimation because underestimated results can cause sunburn to patients due to a prescribed sunbathing time that is too long.

### Model training

We used Adaptive Moment Estimation (ADAM)^49^ as the optimizer. The learning rate was initially set to 0.0005 and iteratively reduced linearly by 1e−7 per epoch. Models were trained for 2000 epochs with a batch size of 256.

## Results

### SurfUVNet model architecture

The task of forecasting in general can be formulated as a problem of finding the best approximation for the relationship between past and future observations. For surface UV radiation, which exhibits an annual seasonal pattern, the profile of next-day UV radiation can be modeled using not only data from previous days but also data from previous years. Here, we adapted an encoder–decoder architecture, which can effectively capture relationship between sequence data, to develop an artificial neural network model for forecasting next-day surface UV radiation. Our model, named SurfUVNet, takes in UV radiation profiles of the past 7, 14, or 21 days through the encoder and passes the encoded information to the decoder. The decoder then takes in the UV radiation profile of the same date as the next day but from last year, combines it with information from the encoder, and then generates the next-day forecast (Fig. 2a). Intuitively, because UV radiation exhibits annual seasonal pattern, our approach models the next-day UV radiation profile as a transformed version of last year’s data and uses recently observed UV pattern to learn the appropriate transformation. Finally, to forecast UV radiation profile further into the future, our approach essentially performs next-day forecast repeatedly via an auto-regressive approach. For example, if we define today as the day *N*, to predict the UV radiation profile for next week, or day *N* + 7, our model first uses data from days *N* − 6, *N* − 5, …, *N* to forecast UV for the day *N* + 1, and then uses the data from days *N* − 5, *N* − 4, …, *N*, and the forecast for the day *N* + 1 to forecast UV for the day *N* + 2, and so on (Fig. 2b).

### Benchmark procedure

**Figure 3:**
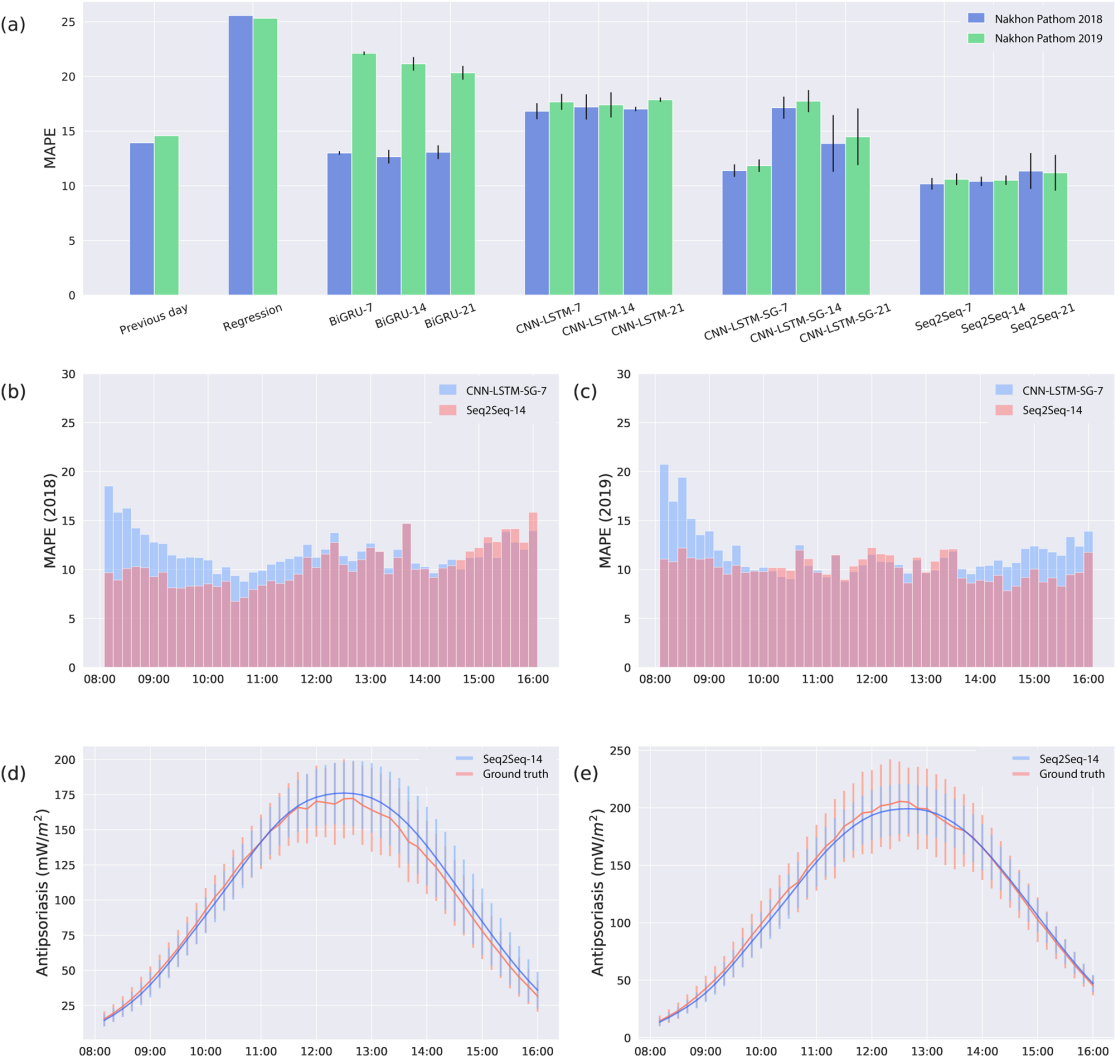
SurfUVNet accurately forecast antipsoriatic irradiance throughout the day. Results on Nakhon Pathom dataset were shown. (**a**) Comparison of the mean absolute percentage errors (MAPE) for the next-day antipsoriatic irradiance forecast between SurfUVNet (Seq2Seq-14) and four benchmark models (see “Methods” section). Previous day model simply predicts next-day’s UV radiation to be the same as today’s. Regression model refers to the regression model based on Earth–Sun distance and total ozone column currently in used by the Thai Meteorological Department^22^. BiGRU is an artificial neural network architecture that is often utilized for time series forecasting. CNN-LSTM, and CNN-LSTM-SG are artificial neural network models that were recently applied to UV forecasting in the energy domain^27^. The tags − 7, − 14, and − 21 designate the length of UV data, in days prior to the forecast date, that were input into each model. (**b**) Distribution of MAPE for the validation set (UV data from 2018) throughout the times of the day. Results for the best performing models, namely CNN-LSTM-SG-7 and SurfUVNet (Seq2Seq-14), are shown. (**c**) A similar plot showing distribution of MAPE for the test set (UV data from 2019). (**d**) Comparison of ground truth UV data and forecasts made by SurfUVNet for the validation set (UV data from 2018). Error bars indicate one-standard deviation ranges. (**e**) A similar plot for the test set (UV data from 2019).

We evaluated the performance of SurfUVNet (also called Seq2Seq-14 here) against four alternative models: a simple model that uses the previous day UV radiation pattern as the prediction, an empirical approach that combined physics knowledge to define the interactions between UV-related factors with regression technique to learn coefficient values, which is currently in used by the Thai Meteorological Department^22^, a CNN-LSTM neural network model developed for solar power forecasting^27^, and an implementation of bidirectional GRU neural network model which is often used in time series forecasting applications. As prior study has shown that the CNN-LSTM model benefits from additional smoothing of UV data from rainy days^27^, we considered two CNN-LSTM model implementations: one without smoothing and one with Savitzky–Golay filter^42^ (denoted by CNN-LSTM and CNN-LSTM-SG in Fig. 3a and Table 1). To fairly compare model performance, the validation and test datasets were subjected to quality filtering to remove days with highly skewed and out-of-range UV irradiance values (see “Methods” section) where all models are expected to perform poorly on. However, it should be noted that this does not mean that our validation and test sets consist of only clear-sky data. The distribution of cloud coverage shows that both datasets contain many days with cloud coverage above 0.2 and up to 0.4 or more (Fig. 1e and f).

**Table 1:** Mean absolute percentage errors (MAPE) of the next-day antipsoriatic irradiance forecasting produced by SurfUVNet and benchmark models on Nakhon Pathom dataset.

Model	Number of parameters	Validation set MAPE (2018, 8AM–4PM)	Test set MAPE (2019, 8AM–4PM)
Previous day model	–	13.93	14.58
Regression model based on Earth–Sun distance and total ozone column^22^	6	25.57	25.32
BiGRU-7	39,165,013	13.00 ± 0.16^b^	22.12 ± 0.33
BiGRU-14	78,158,933	12.66 ± 0.62	21.15 ± 1.20
BiGRU-21	117,152,853	13.07 ± 0.63	20.33 ± 0.47
CNN-LSTM-7^27^	178,077	16.82 ± 0.73	17.67 ± 0.91
CNN-LSTM-14^27^		17.21 ± 1.15	17.40 ± 0.44
CNN-LSTM-21^27^		17.02 ± 0.20	17.87 ± 0.24
CNN-LSTM-SG-7		11.39 ± 0.57	11.84 ± 0.63
CNN-LSTM-SG-14		17.14 ± 1.01	17.74 ± 0.70
CNN-LSTM-SG-21		13.87 ± 2.59	14.48 ± 1.96
Seq2Seq-7	1,627,393	10.18 ± 0.53	10.60 ± 0.34
Seq2Seq-14 (SurfUVNet)		10.41 ± 0.43	10.51 ± 0.41
Seq2Seq-21		11.35 ± 1.64	11.19 ± 0.33

### Next-day antipsoriatic irradiance forecast for Nakhon Pathom dataset

All artificial neural network models were trained using the same UV data from 2011 to 2017 and evaluated on the same UV data from 2018 and 2019 while the regression model based on Earth–Sun distance and total ozone column was fit to UV and ozone data of the same year. All models were trained to forecast next-day antipsoriatic irradiance at 10-min resolution. Furthermore, as past UV radiation profile is a critical input data for artificial neural network models, we tried inputting data from 7, 14, or 21 days prior to the forecast date to explore whether the models benefit from seeing data from more distant past.

Overall, SurfUVNet achieves the best next-day forecasting performance with mean absolute percentage errors (MAPE) of 10.41 and 10.51 on the validation and test sets, respectively (Seq2Seq models in Fig. 3a and Table 1). It should be noted that while the CNN-LSTM-SG model can also reach similar levels of performance (MAPE of 11.39 and 11.84), it is highly sensitive to the length of input UV data. Changing the length of input UV data from 7 days to 14 or 21 days significantly raises the MAPE of CNN-LSTM-SG models to 13.87–17.74. In contrast, the performance of SurfUVNet is stable with respect to the length of the input. Furthermore, SurfUVNet achieves consistent forecasting accuracy throughout the day while the CNN-LSTM-SG model produce significantly higher forecast error during the morning and afternoon hours (8AM–9AM and 2PM–4PM) compared to the middle of the day (Fig. 3b and c). Lastly, comparison of ground truth antipsoriatic irradiance and SurfUVNet’s forecast confirmed that SurfUVNet’s prediction closely mimics the expected bell-shaped pattern of daily UV radiation in both validation and test sets (Fig. 3d and e).

### Next-day downward solar UV irradiance forecast for Tokyo and London datasets

All models were further evaluated on hourly downward solar UV irradiance data obtained from ERA5 for Tokyo, Japan and London, England, which represent different weather regimes from Thailand’s. In contrast to the seasonal cloud coverage pattern at Nakhon Pathom (Fig. 1b), cloud coverage for Tokyo and London fluctuates around 0.2–0.4 year-round (Supplementary Figure 1). Furthermore, day-to-day variation in UV radiation profiles are much higher in Tokyo and London compared to Nakhon Pathom, as indicated by much higher MAPE between today’s and the next day’s UV profiles (Tables 1 and 2, 21.78–35.50 for Tokyo, 18.14–43.57 for London, and 13.93–14.58 for Nakhon Pathom). Overall, SurfUVNet performs competitively, achieving MAPE of 12.72 and 17.74 for the next-day forecast for Tokyo and London datasets, respectively (Table 2). The regression model based on Earth–Sun distance and total ozone column performs much better on these datasets than on Nakhon Pathom’s (Tables 1 and 2, MAPE of 16.52–19.17 on ERA5 compared to 25.52–25.57 on Nakhon Pathom) and only slightly worse than the artificial neural network approaches. Again, it should be noted that the validation and test sets contain many days with considerable cloud coverage (Supplementary Figure 2).

**Table 2:** Mean absolute percentage errors (MAPE) of the next-day antipsoriatic irradiance forecasting produced by SurfUVNet and other models on the ERA5 Tokyo and London datasets.

Model	Tokyo		London	
	Validation set MAPE (2018, 8AM–4PM)	Test set MAPE (2019, 8AM–4PM)	Validation set MAPE (2018, 8AM–4PM)	Test set MAPE (2019, 8AM–4PM)
Previous day model	21.78	35.50	18.14	43.57
Regression model based on Earth–Sun distance and total ozone column^22^	16.68	16.52	18.68	19.17
CNN-LSTM-SG-7	14.75 ± 0.41	15.77 ± 0.33	13.14 ± 0.35	16.27 ± 0.54
CNN-LSTM-SG-14	13.18 ± 0.28	14.99 ± 0.51	12.19 ± 0.19	17.78 ± 0.19
Seq2Seq-14 (SurfUVNet)	11.83 ± 0.44	12.72 ± 0.67	11.54 ± 0.50	17.74 ± 0.19

### Adding weather information does not improve forecasting

**Figure 4:**
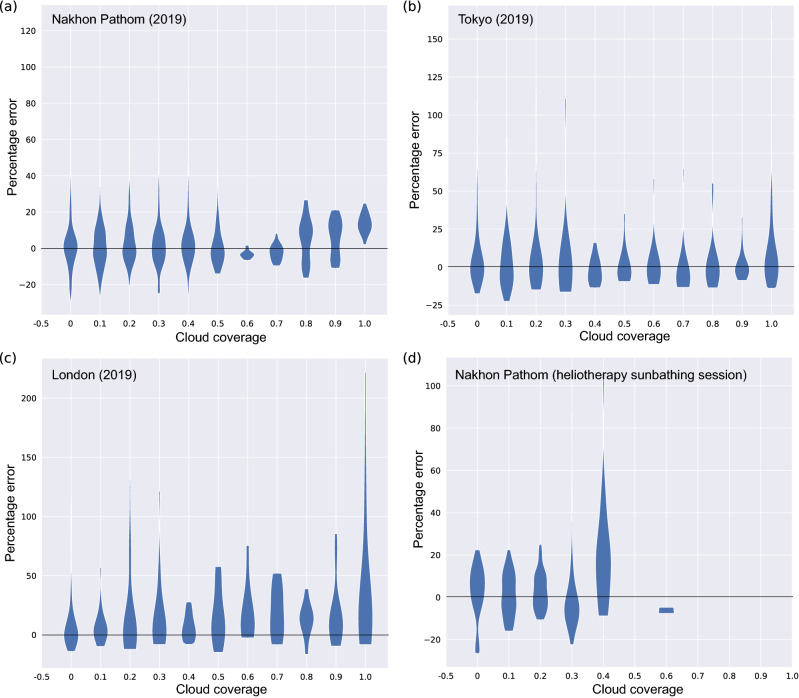
SurfUVNet’s forecast error weakly correlates with cloud coverage. Violin plots showing the distribution of SurfUVNet’s forecast error in 1-h interval with various cloud coverage. Errors on the test sets (UV data from year 2019) are shown. (**a**) Nakhon Pathom dataset. (**b**) Tokyo dataset. (**c**) London dataset. (**d**) Heliotherapy sunbathing sessions planned by photodermatologist at King Chulalongkorn Memorial Hospital. Each data point that constitutes the violin plots correspond to the error between predicted and actual antipsoriatic irradiances that a patient would be exposed to if he or she were to sunbath according to dermatologist’s planning.

As atmospheric conditions can reflect and scatter UV radiation before it reaches the Earth’s surface, we tried incorporating total ozone column, atmospheric aerosol (AOD500), and cloud coverage data into SurfUVNet. However, cloud coverage data contain many missing values that could not be imputed due to the irregularity of the data and had to be excluded from model development. Instead, we used cloud coverage data to evaluate whether SurfUVNet overestimates the amount of UV radiation when the weather is cloudy. This reveals that SurfUVNet’s forecasting errors weakly correlate with cloud condition (Fig. 4, spearman rank correlation = 0.16776, − 0.04546, and 0.20229 for Nakhon Pathom, Tokyo, and London 2019 datasets). For Nakhon Pathom dataset, SurfUVNet’s forecast error stays roughly the same before shifting upward when cloud coverage goes above 0.7 (Fig. 4a). For Tokyo dataset, SurfUVNet’s error is not correlated with cloud coverage at all (Fig. 4b). SurfUVNet’s error shows the clearest correlation with cloud coverage in London dataset (Fig. 4c). Addition of ozone and AOD500 data into SurfUVNet does not improve the performance of the base model that utilizes only UV data (Supplementary Figure 3). The model with ozone and AOD500 data achieves MAPE of 15.33 on the validation set (data from 2017) and MAPE of 13.91 on the test set (data from 2018), while the base model achieves MAPE of 14.32 and 13.60, respectively. This may be because ozone and AOD500 data were collected at lower frequency (hourly vs every 10 min) and at a shorter time period during the day (6AM–6PM vs 5AM–7PM) than UV data. Although data from the early morning and late evening hours where the amount of UV radiation is almost nonexistence should not contribute much to the forecasting of UV radiation during daylight hours, we found that withholding UV data from 6AM to 8AM and 4PM to 6PM from the model slightly raises error from 10.51 to 11.78 MAPE (Wilcoxon signed rank test result is not significant with *p* value = 0.5567). Lastly, to evaluate the impact of uncertainty of next-day ozone and AOD500 on the forecast performance, a variant of SurfUVNet was trained with the actual values of next-day ozone and AOD500. This does not reduce the forecast error (MAPE of 15.70 and 15.50 on the validation and test sets, respectively), indicating that the limitation lies elsewhere.

### Long-term antipsoriatic irradiance forecasting

**Figure 5:**
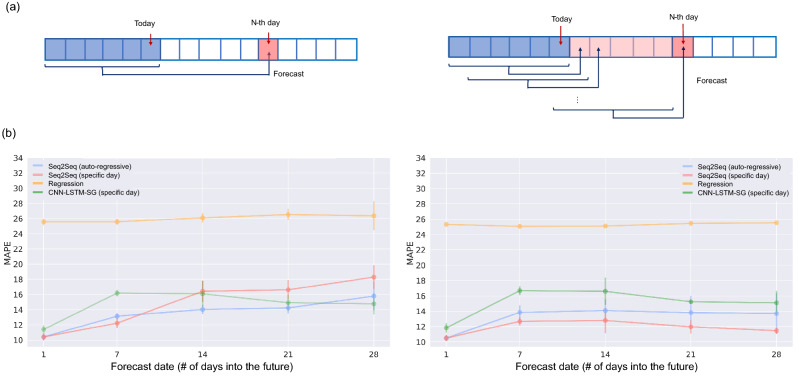
Long-term antipsoriatic irradiance forecasting. Results on Nakhon Pathom dataset were shown. (**a**) Diagram of two approaches for making long-term forecast: developing specific artificial neural network model for making forecast for a specific day that is a certain number of days into the future (left) and autoregressively using the next-day forecast as input for making forecast for the day after that (right). (**b**) Long-term antipsoriatic irradiance forecasting performance for up to 28 days into the future on the validation set (UV data from 2018) and the test set (UV data from 2019). Performance for SurfUVNet, the regression model based on Earth–Sun distance and total ozone column, and the best CNN-LSTM models were shown.

Long-term UV forecasting is essential for heliotherapy applications as it allows clinicians and patients to plan sunbathing schedule in advance and make necessary adjustments to the schedule to achieve the desire UV radiation dosage. We explored two approaches for forecasting antipsoriatic irradiance for up to a month into the future (Fig. 5a). The first approach is to train a collection of artificial neural network models, each making the forecast for a specific date that is a certain number of days into the future. In other words, we trained one model for making the next-day forecast, one model for making the forecast for the day after that, and so on. The second approach is to train a single model for making the next-day forecast and then autoregressively use the next-day forecast as in input to make the forecast for the day after that. Evaluation on Nakhon Pathom 2018–2019 UV datasets showed that the performance of the autoregressive approach is quite stable with average MAPE of 13.70–15.79 for forecasting up to 28 days into the future (Table 3 and Fig. 5b). On the other hand, developing specific models for specific days performs well on the 2019 dataset but poorly on the 2018 dataset (MAPE of 11.46 vs 18.38 for forecasting up to 28 days into the future). We also additionally explored the possibility of training a model that can forecast UV profiles of multiple days at once, but the performances were much worse than the two methods described above (MAPE of 29.49 and 49.69 for forecasting the next 7 days at once on the 2018 and 2019 datasets, respectively). Hence, we decided to choose the autoregressive approach for SurfUVNet. It should be noted that the regression approach based on Earth–Sun distance and ozone information performed poorly on Nakhon Pathom’s UV data even for next-day forecast (Table 1, MAPE of 25.32–25.57).

**Table 3:** Mean absolute percentage errors (MAPE) for long-term antipsoriatic irradiance forecasting for up to 28 days into the future on Nakhon Pathom dataset.

Model	Target day	Validation set MAPE (2018, 8AM–4PM)	Test set MAPE (2019, 8AM–4PM)
Regression model based on Earth–Sun distance and total ozone column^22^	7	25.57	25.07
	14	26.10	25.11
	21	26.53	25.45
	28	26.37	25.53
CNN-LSTM-SG-7 Forecast specific date	7	16.18 ± 0.42	16.68 ± 0.59
	14	16.09 ± 1.70	16.60 ± 1.77
	21	14.91 ± 1.08	15.24 ± 0.29
	28	14.76 ± 1.39	15.09 ± 1.56
CNN-LSTM-SG-7 Auto-regressive	7	18.56 ± 2.65	17.41 ± 2.70
	14	20.60 ± 2.31	19.49 ± 2.92
	21	22.51 ± 1.97	21.37 ± 1.83
	28	24.45 ± 1.60	22.62 ± 0.98
CNN-LSTM-SG-14 Forecast specific date	7	16.69 ± 0.92	16.73 ± 1.16
	14	14.82 ± 0.63	15.49 ± 1.43
	21	15.96 ± 0.99	16.18 ± 0.46
	28	15.46 ± 0.71	15.55 ± 1.14
CNN-LSTM-SG-14 Auto-regressive	7	17.31 ± 0.45	17.77 ± 0.55
	14	17.20 ± 0.47	19.16 ± 0.65
	21	17.13 ± 0.50	20.71 ± 0.57
	28	17.00 ± 0.50	20.83 ± 0.97
Seq2Seq-14 (SurfUVNet) Forecast specific date	7	12.21 ± 0.65	12.68 ± 0.53
	14	16.42 ± 1.40	12.80 ± 1.64
	21	16.61 ± 1.29	11.97 ± 0.88
	28	18.28 ± 1.57	11.46 ± 0.38
Seq2Seq-14 (SurfUVNet) Auto-regressive	7	13.13 ± 0.41	13.86 ± 0.91
	14	14.03 ± 0.62	14.09 ± 1.49
	21	14.22 ± 0.74	13.83 ± 2.20
	28	15.79 ± 1.90	13.70 ± 2.65

## Discussion

We have developed SurfUVNet, an artificial neural network model for predicting surface UV radiation that achieves around 10% error for next-day forecast and 13–16% error for 7-day up to 4-week forecast. This affirms that quantitative UV forecast is appropriate for heliotherapy applications, which tolerate up to 10–25% error level. SurfUVNet’s performance is competitive on UV data from multiple regions, Thailand, Japan, and England, and on both antipsoriatic and downward irradiance. Hence, SurfUVNet can be adapted for forecasting other useful UV action spectra such as vitamin D production and erythemal UV index as well. In fact, our model can even be trained to forecast antipsoriatic irradiance from input erythemally-weighted UV data from a UV Biometer instrument with a small performance reduction (data now shown). This capability is necessary for establishing a national heliotherapy network in Thailand because there is only one full-spectrum UV sensor located in the central region of the country while the rest of the country is covered by a network of UV Biometers.

A key limitation of artificial neural network is that it tends to overfit to the training dataset and does not generalize well to other datasets that come from different distributions. In the context of UV forecasting, this dictates that the model must be retrained with data from particular weather station in order to be usable for that geographic region. Indeed, the accuracy of each model varies by 5–6% across the three geographical regions, Thailand, Japan, and England and even across 2018 and 2019 in the case of London dataset (Tables 1 and 2). For the case of London dataset, comparison of UV profiles between consecutive days in 2019 showed an extremely high average variation of 43.57%. The discrepancy in performance of the regression model based on Earth–Sun distance and total ozone column developed by the Thai Meteorological Department^22^ between Nakhon Pathom and ERA5 datasets (25% error on Nakhon Pathom and 16–19% error on ERA5^41^ datasets) could be attributed to the fact that ERA5 data, which contain more detailed ozone measurements (hourly compared to daily) and were computationally interpolated, are likely to be more easily fitted by regression.

The fact that SurfUVNet’s forecast error only weakly correlates with cloud coverage (Fig. 4) is unexpected but may be explained by the fact that cloud coverage in Nakhon Pathom exhibits clear seasonal pattern (Fig. 1b) and that the UV radiation profiles are stable over consecutive days (Table 1, MAPE of 13.94–14.58 for previous day model). On a geographical region with highly variable weather condition, such as London in 2019, artificial neural network models’ performance drop significantly (Table 2) and the error of SurfUVNet exhibits higher correlation with cloud coverage (Fig. 4c). Hence, artificial neural network models seem to be able to exploit seasonal weather pattern and day-to-day variation to achieve good performance without relying on explicit cloud coverage information. This capability of the model to extract seasonal patterns may also explain why addition of ozone and AOD500 information did not improve the performance of SurfUVNet (Supplementary Figure 3), particularly as AOD500 level at Nakhon Pathom closely follows the same seasonal pattern as cloud coverage (Fig. 1d).

We explored two approaches for forecasting long-term UV radiation. Initially, we expected that developing a specific model for making the forecast for a specific date a certain number of days into the future would yield better performance than an autoregressive approach which use the next-day forecast as input for making the forecast for the day after because forecasting errors would accumulate through autoregressive steps. However, the models for specific date seem to overfit the training data, performing well on the 2019 dataset but poorly on the 2018 dataset (Table 3, 11.46% vs 18.28% error for forecasting up to 28 days into the future). In contrast, the autoregressive approach performs more consistently (13.70% and 15.79% error). An explanation for the overfitting of the model trained for specific date may be because the relationship between today’s and next week’s UV radiation profiles is so weak that the models learn mostly patterns that are specific to the training dataset. The poor performance of models for multi-day forecast (29.49–49.69% error for 7-day forecast) is likely due to the sheer number of outputs that the models must optimize. To make a 7-day forecast at 10-min resolution, the model has to output 595 values. From these results, we recommend the autoregressive approach for making long-term UV forecast with SurfUVNet.

To prospectively examine whether SurfUVNet’s performance is sufficient for heliotherapy applications, we asked photodermatologist at King Chulalongkorn Memorial Hospital to plan a 3-month sunbathing course based on SurfUVNet’s output and then compared their schedule with the ground truth antipsoriatic irradiances of the same time interval. This reveals that the error in antipsoriatic dose that the patient would receive by following the clinician’s sunbathing protocol remains well within the acceptable 10–25% up to 0.3 cloud coverage (Fig. 4d, MAPE of 11.23). A possible solution for accounting for weather effects on UV radiation that we are exploring is to have each patient carry a portable UV sensor or a smartphone equipped with light sensor and use that data to adjust SurfUVNet’s forecast in real-time.

## Supplementary Information

Supplementary Information.
