# Supplementary material for: Accurate surface ultraviolet radiation forecasting for clinical applications with deep neural network
Source: Sci Rep. 2021 Mar 3;11:5031. doi: 10.1038/s41598-021-84396-2 (PMC7930112; doi:10.1038/s41598-021-84396-2)
Supplement: Supplementary file 1 — Supplementary Information. [file 41598_2021_84396_MOESM1_ESM.pdf]

Title: Accurate Surface Ultraviolet Radiation Forecasting for Clinical Applications with Deep Neural Network

R. Raksasat[1,2], P. Sri-iesaranusorn[1,3], J. Pemcharoen[4], P. Laiwarin[5], S. Buntoung[5], S. Janjai[5], E. Boontaveeyuwat[4], P. Asawanonda[4], S. Sriswasdi[2,6,*], E. Chuangsuwanich[1,2,*]

Supplementary figures

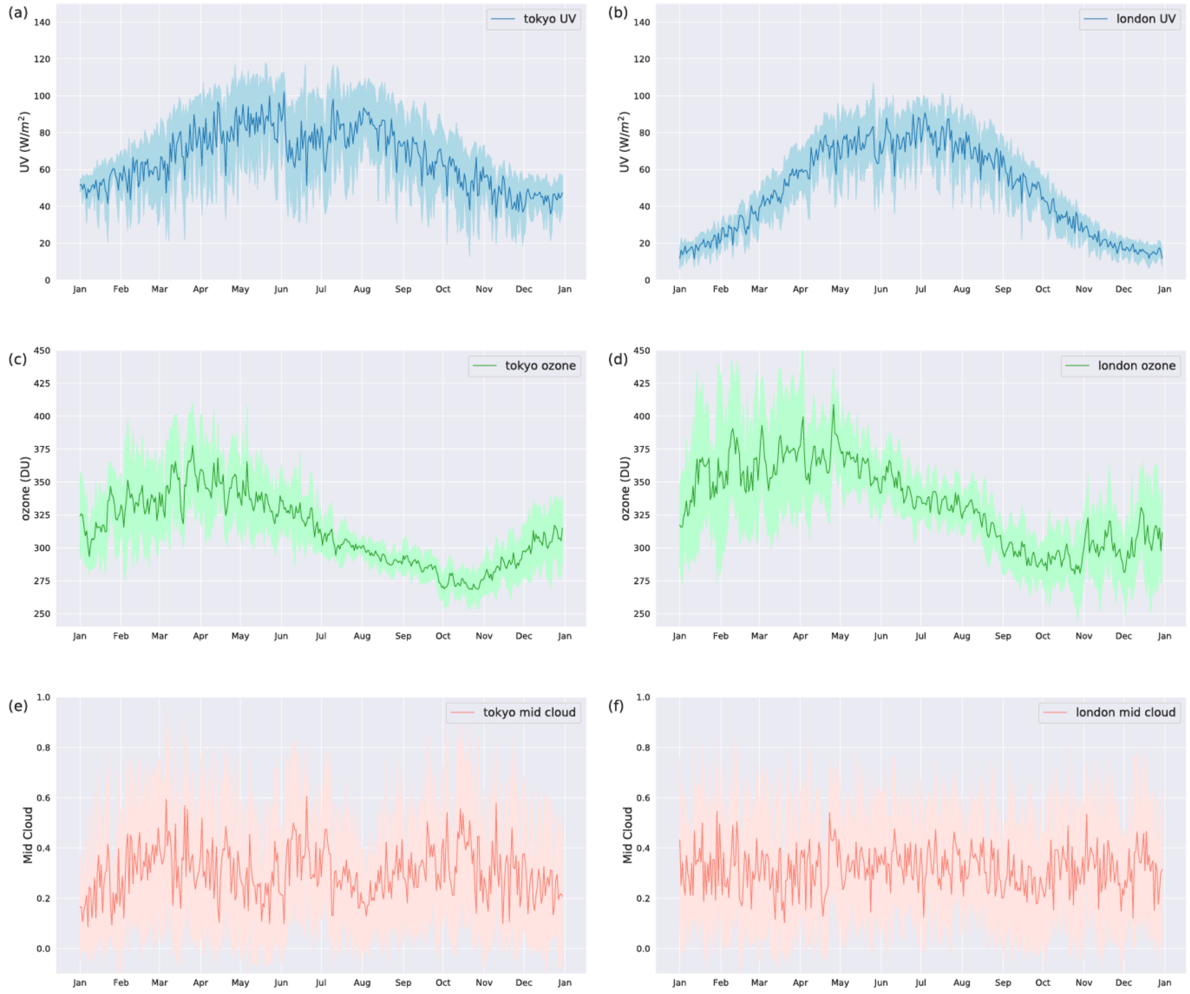

Supplementary Figure 1: Characteristics of UV and weather conditions at Tokyo. Japan and London, England. Daily maximums from ERA5 datasets are shown. Dark lines indicate the average across 2009-2017. Shaded areas indicate the ±1 standard deviation range. (a) Annual downward surface UV irradiance at Tokyo. (b) Annual downward surface UV irradiance at London. (c) Annual total ozone column at Tokyo. (d) Annual total ozone column at London. (e) Annual cloud coverage at Tokyo. (f) Annual cloud coverage at London.

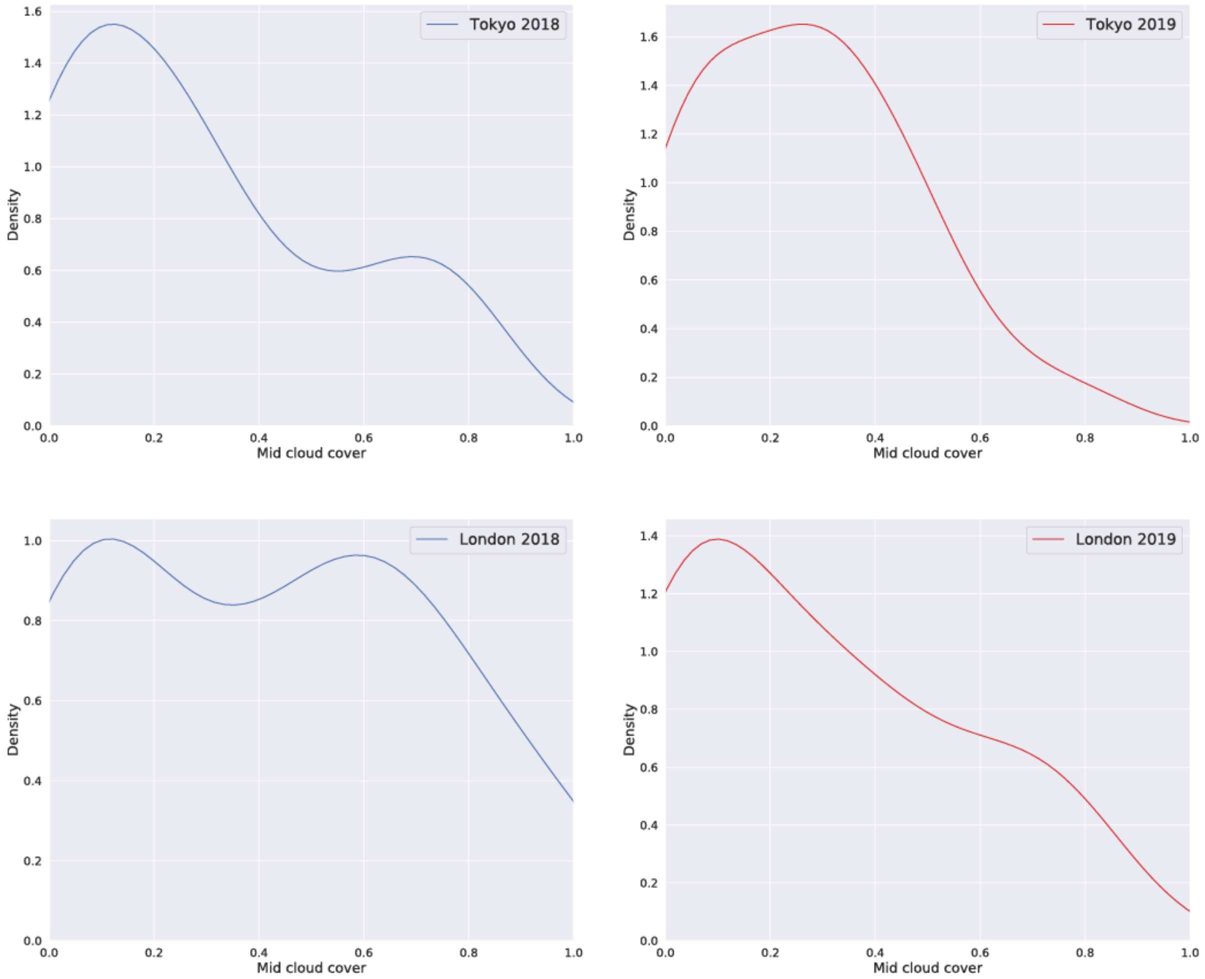

Supplementary figure 2: The distribution of cloud coverage in ERA5 validation and test sets. Mid cloud coverage data are shown. (a) Cloud coverage distribution for validation set (UV data from year 2018) for Tokyo. (b) Test sets (UV data from year 2019) for Tokyo. (c) Validation set (UV data from year 2018) for London. (d) Test sets (UV data from year 2019) for London.

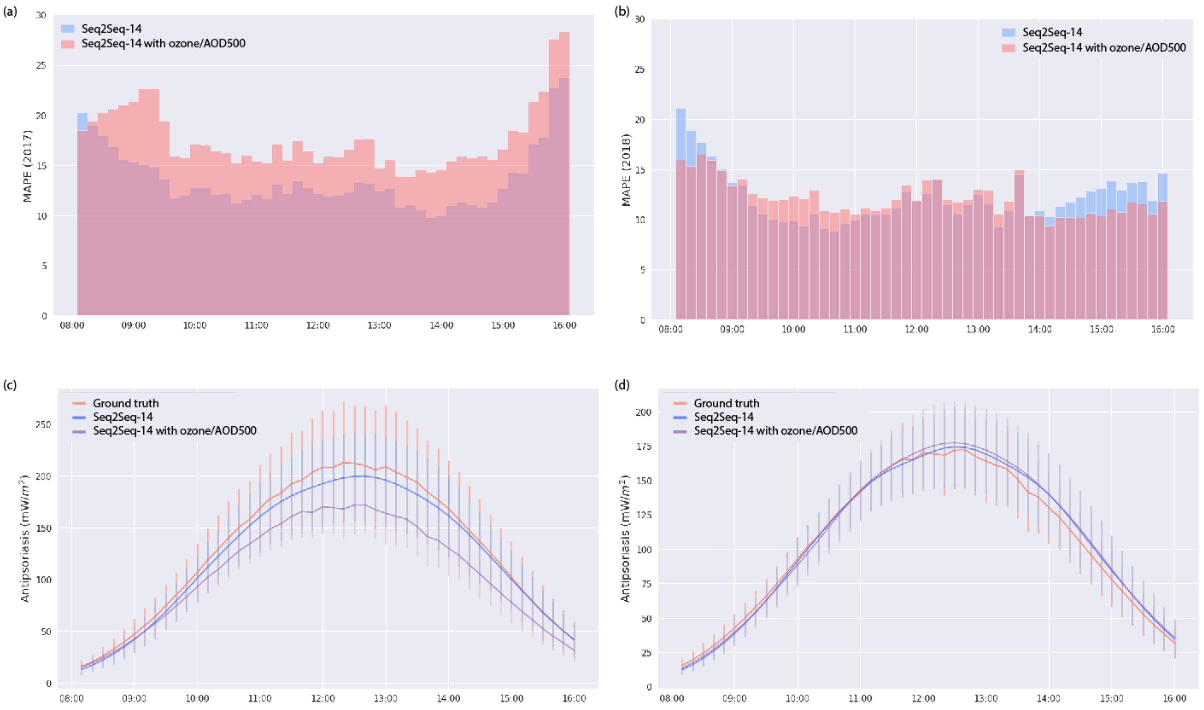

Supplementary figure 3: Comparison of SurfUVNet performance with and without ozone and AOD500 as input. It should be noted that as AOD500 data at Nakhon Pathom for the year 2019 were not available, UV data from year 2017 were set as the validation set and UV data from year 2018 were set as the test set here. (a) Distribution of MAPE for the validation set (UV data from 2017) throughout the times of the day. (b) A similar plot showing distribution of MAPE for the test set (UV data from 2018). (c) Comparison of ground truth UV data and forecasts made by SurfUVNet for the validation set (UV data from 2017). Error bars indicate one-standard deviation ranges. (d) A similar plot for the test set (UV data from 2018).
